# Impact of Aneurysmal Subarachnoid Hemorrhage Severity on Contrast Media Arrival Time in Head Computed Tomography Angiography

**DOI:** 10.7759/cureus.80287

**Published:** 2025-03-09

**Authors:** Kazutoshi Tsunou

**Affiliations:** 1 Department of Central Radiology, Japanese Red Cross Okayama Hospital, Okayama, JPN

**Keywords:** 3dct, bolus tracking, cerebral aneurysm, cerebral vessel cta, endovascular treatment, hunt & hess grade

## Abstract

Aim

This study aims to evaluate the effect of subarachnoid hemorrhage (SAH) severity on contrast media (CM) arrival time in head computed tomography angiography (CTA) at SAH onset.

Method

A total of 67 patients who underwent head CTA were evaluated; 41 patients developed SAH (SAH group), and 26 patients had suspected unruptured cerebral aneurysms (non-SAH group). The patients of the SAH group were divided into mild (grades I-III), semi-severe (grade IV), and severe (grade V) groups according to Japanese guidelines. CM arrival time was measured for each case.

Results

The CM arrival time increased with SAH severity. The semi-severe and severe groups exhibited significantly longer CM arrival times compared to the non-SAH group (non-SAH: 11.1 ± 2.03, mild: 13.2 ± 2.97, semi-severe: 15.8 ± 3.45, severe: 16.6 ± 3.40).

Conclusion

The CM arrival time increases with SAH severity in head CTA at SAH onset. Therefore, it is important for operators to be aware of the possibility of slower-than-usual timing in severe cases of SAH.

## Introduction

Rupture of a cerebral aneurysm (AN) is the primary etiology of non-traumatic subarachnoid hemorrhage (SAH) [1]. Imaging of cerebral AN is mainly performed using catheter-based digital subtraction angiography (DSA), which can appropriately visualize the location and composition of AN, entry and exit vessels, and the relationship of AN with nearby vessels [1,2]. The performance of multidetector row computed tomography (MDCT) has improved in recent years. The detection of cerebral AN with head computed tomography angiography (CTA), which is obtained by rapid injection of contrast media (CM), has been reported to be as good as or better than that of DSA and is widely used [3-6]. Head CTA can diagnose 80-90% of cerebral ANs, and although it has limitations in detecting ANs < 2 mm, it can observe cerebral AN from any direction; therefore, it has a wide range of applications, including preoperative simulation and explanation to patients as well as morphological evaluation [1,7-9].

Non-contrast head computed tomography (CT) serves as the diagnostic modality to ascertain the presence of subarachnoid blood [1-3,10]. When SAH is identified via non-contrast head CT, head CTA should be performed immediately. Nevertheless, in severe cases, intracranial hypertension, along with potential complications such as takotsubo cardiomyopathy and lethal arrhythmias, may impede CM arrival in the brain, hindering the acquisition of suitable cerebrovascular CT attenuation. Accurate scan timing is critical for obtaining CTA, but the influence of SAH severity on CM arrival time remains ambiguous. Elucidating the influence of these pathological conditions is essential in determining optimal scan time.

There are limited studies on head CTA at SAH onset that address scanning timing. Clarifying the relationship between SAH severity and CM arrival time is a crucial first step in developing an optimal scanning protocol. This study aimed to evaluate the effect of SAH severity on CM arrival time in head CTA at SAH onset.

## Materials and methods

Patients

This study enrolled 41 consecutive patients who experienced SAH between April 2016 and March 2020 and subsequently underwent head CTA to detect cerebral AN (SAH group). A neurosurgeon with 10 years of experience with patients in the SAH group confirmed that the clinical diagnosis of SAH severity was faithful to the Hunt and Hess grading system [10]. The "Japan Stroke Society Guideline 2021 for the Treatment of Stroke" has different treatment strategies for each SAH severity [11]. It is reasonable to perform open surgery or endovascular treatment to prevent rebleeding within 72 hours after the onset in severity grades I to III patients without any limitations of age, systemic complications, or difficulty of treatment. In patients with severity grade IV, the adequacy of open surgery or endovascular treatment may be determined, considering the patient's age and the location of the aneurysm. In patients with severity grade V, open surgery or endovascular treatment may be considered when their neurological conditions improve after admission. Patients in the SAH group were divided into the mild (grades I-III), semi-severe (grade IV), and severe (grade V) groups.

Additionally, 26 patients who underwent head CTA between June 2017 and March 2018 with suspected unruptured cerebral AN and were scanned using the CTA protocol for cerebral AN scrutiny (non-SAH group) were included. Given that CM injection is weight-dependent, patients whose weight was undocumented at the time of scanning were excluded from this study. The SAH group consisted of two grade I cases, 11 grade II cases, six grade III cases, 13 grade IV cases, and nine grade V cases.

Scan parameters and CM injection method

CT scans were performed using a 64-slice Aquilion64CX (Canon Medical Systems, Tochigi, Japan). Nonionic CM with iodine concentrations of 300-370 mg/ml were selectively used depending on body weight (Iohexol 300, Iohexol 350, Iopamidol 370; Daiichi Sankyo Pharmacy, Tokyo, Japan). CM was injected at 375 mg of iodine/kg of body weight in 15 seconds using a motorized injector (Dual Shot GX; Nemoto Kyorindo, Tokyo, Japan), followed by administering 30 ml of saline at the same rate. Computer-aided bolus tracking technology (Real Prep, Canon Medical Systems, Tochigi, Japan) was used for arterial phase studies. Real Prep parameters were as follows: tube voltage, 100 kVp; tube current, 50 mA; detector collimation, 4 x 1.0 mm; FOV (field of view), 240 mm. The CM arrival was monitored at the cervical vertebrae level (C1-C2), and the radiologic technologist visually confirmed when the CM reached the internal carotid artery (ICA) and triggered the scan manually.

Measurement of CM arrival time

**Figure 1:**
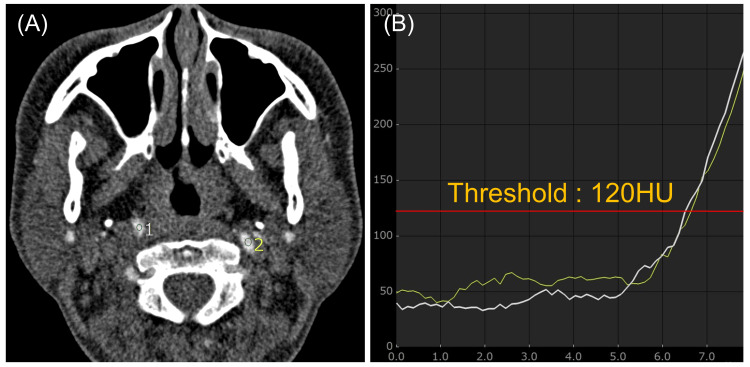
Method of measuring contrast media arrival time A and B: Time enhancement curves were obtained by placing regions of interest on images obtained from computer-aided bolus tracking technology (Real Prep, Canon Medical Systems). Contrast media (CM) arrival time was defined as the time from the start of CM injection until the mean computed tomography (CT) attenuation of the internal carotid artery (ICA) reached 120 Hounsfield units.

A radiologic technologist with 19 years of experience evaluated all patients using SYNAPSE VINCENT Ver6.4 (Fujifilm Medical, Tokyo, Japan). A region of interest of 3.29-6.79 mm^2^ was placed within the ICA based on Real Prep data reconstructed at 0.2-second intervals, and a time enhancement curve was created. The CM arrival time in this study was defined as the time from the start of CM injection until the mean CT attenuation of the ICA reached 120 Hounsfield units (Figure 1).

Statistical analysis

Statistical analyses were performed using EZR (Saitama Jichi Medical University, Saitama, Japan) [12]. Fisher's exact test was used for nominal variables. Continuous data were tested for normality using the Shapiro-Wilk test. The Mann-Whitney U test or Welch t-test was applied depending on variance assumptions. Differences in CM arrival times among groups were evaluated using the Tukey-Kramer multiple comparisons test. A p-value < 0.05 was considered statistically significant.

## Results

Patient characteristics

Patient characteristics of both groups are shown in Table 1. There were no significant differences in sex, age, weight, and BMI between the two groups.

**Table 1:** Patient characteristics of the subarachnoid hemorrhage (SAH) and non-SAH groups Data are expressed as mean ± standard deviation. BMI: body mass index

Parameters	SAH Group	Non-SAH Group	Statistical Analysis	p-value
Patients (n)	41	26	-	-
Sex (male/female)	17/24	7/19	Fisher's exact test	0.30
Age (years)	65.2 ± 16.4	65.3 ± 16.0	Mann-Whitney U test	0.90
Body weight (kg)	55.8 ± 11.1	55.9 ± 8.6	Student t-test	0.99
BMI (kg/m^2^)	22.5 ± 4.4	22.8 ± 2.6	Welch t-test	0.67

Comparison of CM arrival time

**Figure 2:**
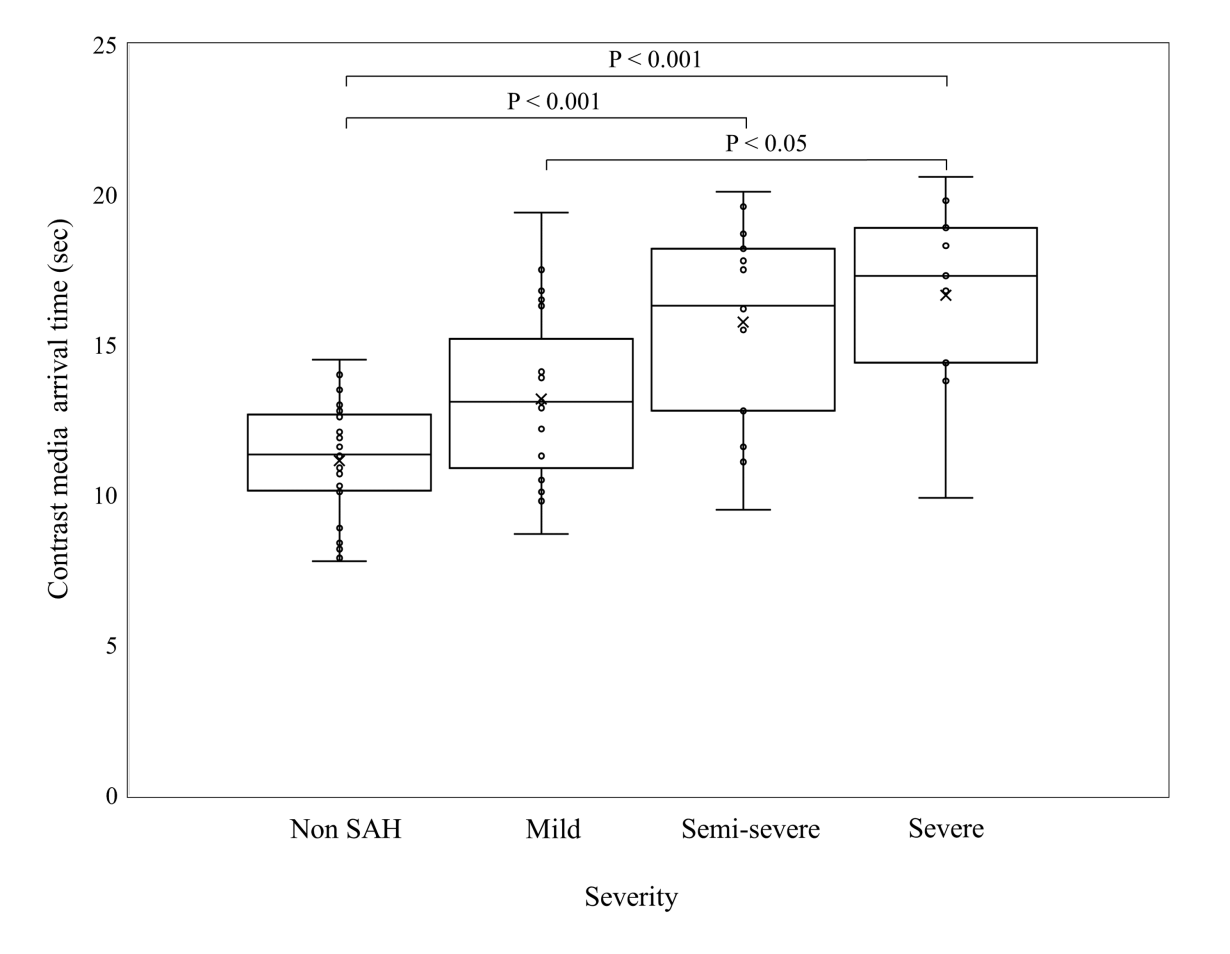
Comparison of contrast media arrival time The semi-severe and severe groups exhibited significantly longer CM arrival times compared to the non-subarachnoid hemorrhage (SAH) group.

The CM arrival times are shown in Figure 2. The CM arrival time increased with SAH severity. The semi-severe and severe groups exhibited significantly longer CM arrival times compared to the non-SAH group (non-SAH: 11.1 ± 2.03, mild: 13.2 ± 2.97, semi-severe: 15.8 ± 3.45, severe: 16.6 ± 3.40).

Representative case

**Figure 3:**
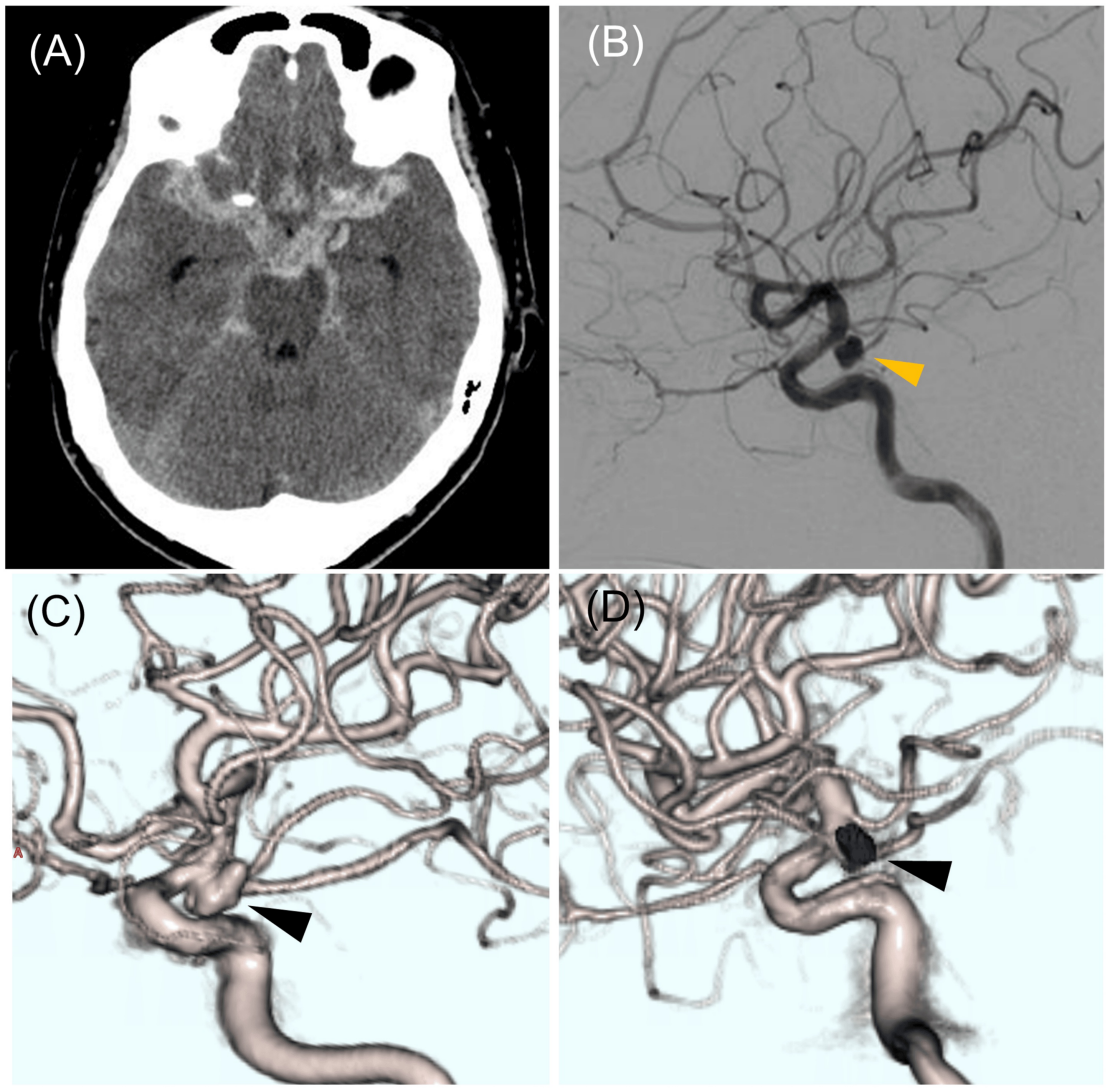
A case of subarachnoid hemorrhage due to the rupture of a left internal carotid artery aneurysm A left ICA aneurysm (AN) was found in a female in her 50s (Hunt and Hess grade IV) on head CT angiography, and endovascular treatment was performed. (A) CT image at the time of the visit. (B) Digital subtraction angiography image. The yellow arrowhead indicates AN. (C) Three-dimensional rotational angiography. The black arrowhead indicates AN; (D) Three-dimensional rotational angiography after coil embolization. The clump indicates coils (black arrowhead).

Figure 3 illustrates a case of SAH. AN of the left ICA was found in a female in her 50s (Hunt and Hess grade IV) on head CTA, and endovascular treatment was performed. The patient developed takotsubo cardiomyopathy at SAH onset, but cardiologists were able to confirm that cardiac function had improved on the sixth day after surgery. On the eighth day after surgery, a head CTA was performed to evaluate cerebral vasospasm. The CM arrival time was 20.1 seconds at the onset of SAH and 12.1 seconds on the eighth day after surgery.

## Discussion

This study evaluated the effect of SAH severity on CM arrival time in head CTA at SAH onset. Our data showed that CM arrival time increased with SAH severity.

The high-speed scanning capability of MDCT offers precise arterial visualization contingent upon accurate scan timing [13,14]. If scanning is performed too early, image acquisition may conclude before peak arterial enhancement is reached. Conversely, if scanning is performed too late, the peak arterial enhancement may be missed, and arterial visibility may be reduced due to venous opacity. Several factors influence scan timing, including technical parameters (e.g., CM injection speed and volume) and patient-specific factors (e.g., body weight, height, and cardiac output) [15,16]. Among these, cardiac output and body weight are particularly important. There is an inversely proportional relationship between the magnitude of the enhancement and body weight. The heavier the patient, the greater the volume of blood and other tissues; thus, the higher the dose of contrast required.

Iodine load is defined as the amount of iodine administered per body weight of the patient. It is usually expressed as milligrams of iodine per kilogram of body weight (mgI/kg). The iodine load is the key parameter to consider in studies that aim to achieve visceral enhancement [16]. Therefore, in this study, CM concentrations and injection speed were adjusted based on the patient body weight, and patients whose weight was unknown at the time of scanning were excluded. As cardiac output decreases, blood circulation slows, resulting in delayed CM arrival [15,16]. Compared to the non-SAH group, the semi-severe and severe SAH groups exhibited significantly prolonged CM arrival times. Cardiac dysfunction, commonly observed in severe SAH cases, likely contributes to this delay [15-17]. Additionally, cerebral hypoperfusion due to SAH may further exacerbate delayed CM arrival [18].

It should be emphasized that the head CTA presented in the representative SAH case deviates from that in the non-SAH group because it was performed after the procedure to prevent rebleeding. However, CM arrival time showed a difference of eight seconds. The patient had developed takotsubo cardiomyopathy, and her cardiac function was impaired. Takotsubo cardiomyopathy induced by SAH tends to occur with higher SAH severity and is known to be more common in women [17,19]. In cases where cardiac dysfunction is evident during head CTA, prolonged CM arrival time should be anticipated, and scan timing should be adjusted accordingly.

Currently, bolus tracking technology is most frequently used in clinical practice to help monitor individual CM arrival and adjust scan timing [20]. The bolus tracking technique monitors CM arrival at a predetermined level and initiates scanning with a fixed scan delay after CM arrival. The optimal scan delay for head CTA is six to eight seconds after the CM reaches the Willis artery ring in the absence of cerebrovascular disease [13,14]. However, adapting these scan delays at SAH onset may be difficult. This is due to the fact that, with the onset of SAH, the optimal scan delay may also be longer because CM arrival time is longer depending on SAH severity. In other words, it is necessary to construct a scan protocol with a more adjusted delay than usual at SAH onset. At our facility, the operator adjusted the scan delay time by manually triggering the scan. However, this method is highly operator-dependent. Further investigation is needed to validate the optimal scan delay at SAH onset independent of the operator.

Our study had several limitations. First, because this was a retrospective study conducted in a clinical setting, we did not compare SAH and non-SAH using the same group of patients. The non-SAH group consists of patients with cerebral AN, whereas the SAH group comprises patients with ruptured cerebral AN. This distinction introduces a potential bias regarding factors that contribute to aneurysm rupture. However, it would have been difficult for ethical reasons to obtain images of patients with SAH before SAH onset. Second, the CM arrival time measured in this study was obtained using arterial tracking data and may not fully reflect actual CM arrival delays in the cerebral circulation. Monitoring CM arrival in the brain may yield different results. Third, we did not evaluate individual intracranial pressure or cardiac function in SAH patients. Additional studies are needed to determine the extent to which cerebral hypoperfusion or cardiac dysfunction due to SAH affects CM arrival time.

## Conclusions

Head CTA is useful in the search for cerebral AN. Although accurate scan timing is critical for CTA, there are no clinical studies on the effect of SAH severity on CM arrival time. CM arrival time increases with SAH severity in head CTA at SAH onset. In particular, grades Ⅳ and Ⅴ exhibited significantly longer CM arrival times compared to the normal. Therefore, it is important for operators to be aware of the possibility of slower-than-usual timing in severe cases of SAH.
